# An integrative modular approach to systematically predict gene-phenotype associations

**DOI:** 10.1186/1471-2105-11-S1-S62

**Published:** 2010-01-18

**Authors:** Michael R Mehan, Juan Nunez-Iglesias, Chao Dai, Michael S Waterman, Xianghong Jasmine Zhou

**Affiliations:** 1 Program in Computational Biology, Department of Biological Sciences, University of Southern California, Los Angeles CA 90089, USA; 2 School of Computer, Wuhan University, Wuhan 430079, PR China

## Abstract

**Background:**

Complex human diseases are often caused by multiple mutations, each of which contributes only a minor effect to the disease phenotype. To study the basis for these complex phenotypes, we developed a network-based approach to identify coexpression modules specifically activated in particular phenotypes. We integrated these modules, protein-protein interaction data, Gene Ontology annotations, and our database of gene-phenotype associations derived from literature to predict novel human gene-phenotype associations. Our systematic predictions provide us with the opportunity to perform a global analysis of human gene pleiotropy and its underlying regulatory mechanisms.

**Results:**

We applied this method to 338 microarray datasets, covering 178 phenotype classes, and identified 193,145 phenotype-specific coexpression modules. We trained random forest classifiers for each phenotype and predicted a total of 6,558 gene-phenotype associations. We showed that 40.9% genes are pleiotropic, highlighting that pleiotropy is more prevalent than previously expected. We collected 77 ChIP-chip datasets studying 69 transcription factors binding over 16,000 targets under various phenotypic conditions. Utilizing this unique data source, we confirmed that dynamic transcriptional regulation is an important force driving the formation of phenotype specific gene modules.

**Conclusion:**

We created a genome-wide gene to phenotype mapping that has many potential implications, including providing potential new drug targets and uncovering the basis for human disease phenotypes. Our analysis of these phenotype-specific coexpression modules reveals a high prevalence of gene pleiotropy, and suggests that phenotype-specific transcription factor binding may contribute to phenotypic diversity. All resources from our study are made freely available on our online Phenotype Prediction Database [1].

## Background

A major goal of modern genetics is to determine which genes are associated with which human phenotypes. Over the course of the last few decades, studies uncovering the basis for Mendelian diseases have been extremely successful, typically identifying causal mutations in single genes [2]. However, most human phenotypes, e.g. complex diseases such as cancer or neurological diseases, are controlled by multiple genes, each of which has a minor contribution to the disease phenotype [3]. Therefore, to effectively identify genes that are related to complex phenotypes, the approach must consider groups of genes rather than studying genes in isolation. Numerous methods have been developed for identifying gene modules from protein-protein interaction (PPI) networks [4], metabolic networks [5], or transcription-regulatory networks [6], however these network data are often lack human phenotype-specific information.

In this study, we identified gene modules that are specifically coexpressed in datasets that study particular human phenotypes. Identifying phenotype-specific modules in human is far more difficult than in model organisms because the phenotypes can only be observed rather than working directly with the biological pathways that define them. Ideally, these biological pathways should be reverse-engineered from data taken from individuals that display particular phenotypes. Public repositories of microarray data are a valuable resource for this type of analysis because they contains hundreds of well annotated expression datasets that span a wide variety of phenotypic conditions. It is known that identifying co-expression modules frequently occurring across multiple microarray datasets significantly enhances the signal to noise ratio [7-9]. Here, we identified co-expression modules that are present recurrently and specifically in datasets of one phenotype by using the remaining datasets as a background.

Using these phenotype-specific coexpression modules, we performed a systematic prediction of gene-phenotype associations by integrating three data sources: previously known associations derived from literature text mining, Gene Ontology annotations, and protein-protein interactions. A previous study designed an approach to identify gene modules in human PPI networks and used them to predict novel gene-phenotype associations [10]. Our approach differs from this in that it integrates hundreds of microarray datasets in parallel to identify modules, and then superimposes protein-protein interactions as well as phenotype and functional annotations to make predictions.

The systematic annotation of gene-phenotype associations provides us with the opportunity to perform the first global analysis of gene pleiotropy in human. Gene pleiotropy has the potential to explain the vast human phenotypic diversity, considering that the number of human genes is far fewer than originally anticipated [11]. Several large scale studies of pleiotropy have been performed on model organisms such as yeast [12,13] and C. elegans [14]. In humans however, pleiotropy is often only recognized following the in-depth analysis of a single gene or gene family [15-17]. To our knowledge, no comprehensive determination or prediction of which genes exhibit pleiotropic behavior throughout the entire human genome has been previously performed. Based on our modular approach, we have defined a novel concept, *modular pleiotropy*, as the pleiotropic behavior of genes resulting from their presence in their modules, as we have shown that changes in module membership can define a gene's pleiotropic behavior. To further understand the underlying mechanisms of phenotypic diversity, we utilized the rapid accumulation of ChIP-chip datasets, measured under various phenotypic conditions, and tested whether the phenotype specificity of our modules, as well as specific instances pleiotropy, could be attributed to dynamic phenotype-specific gene regulation.

By integrating 338 human microarray datasets representing 283 phenotypes, we have identified 193,145 phenotype-specific modules. We subsequently predicted 6,558 novel gene-phenotype associations covering 3,183 genes, and showed that 40.9% of genes are associated with multiple phenotypes, and can thus be considered pleiotropic. We collected 77 Chip-chip datasets, annotated them with matching phenotypes, and confirmed that dynamic transcriptional regulation is an important force driving the formation of phenotype-specific modules. Our module-based approach has the advantage of not only predicting pleiotropy, but also suggesting *how *a gene is pleiotropic, exerting different phenotypic functions in different transcriptional and regulatory contexts.

We have provided all the data from our study, including the gene phenotype database we constructed via text mining, our phenotype-specific modules, and our novel phenotype predictions, in our online Phenotype Prediction Database [1].

## Results and discussion

### Systematic annotation of gene-phenotype association

**Figure 1:**
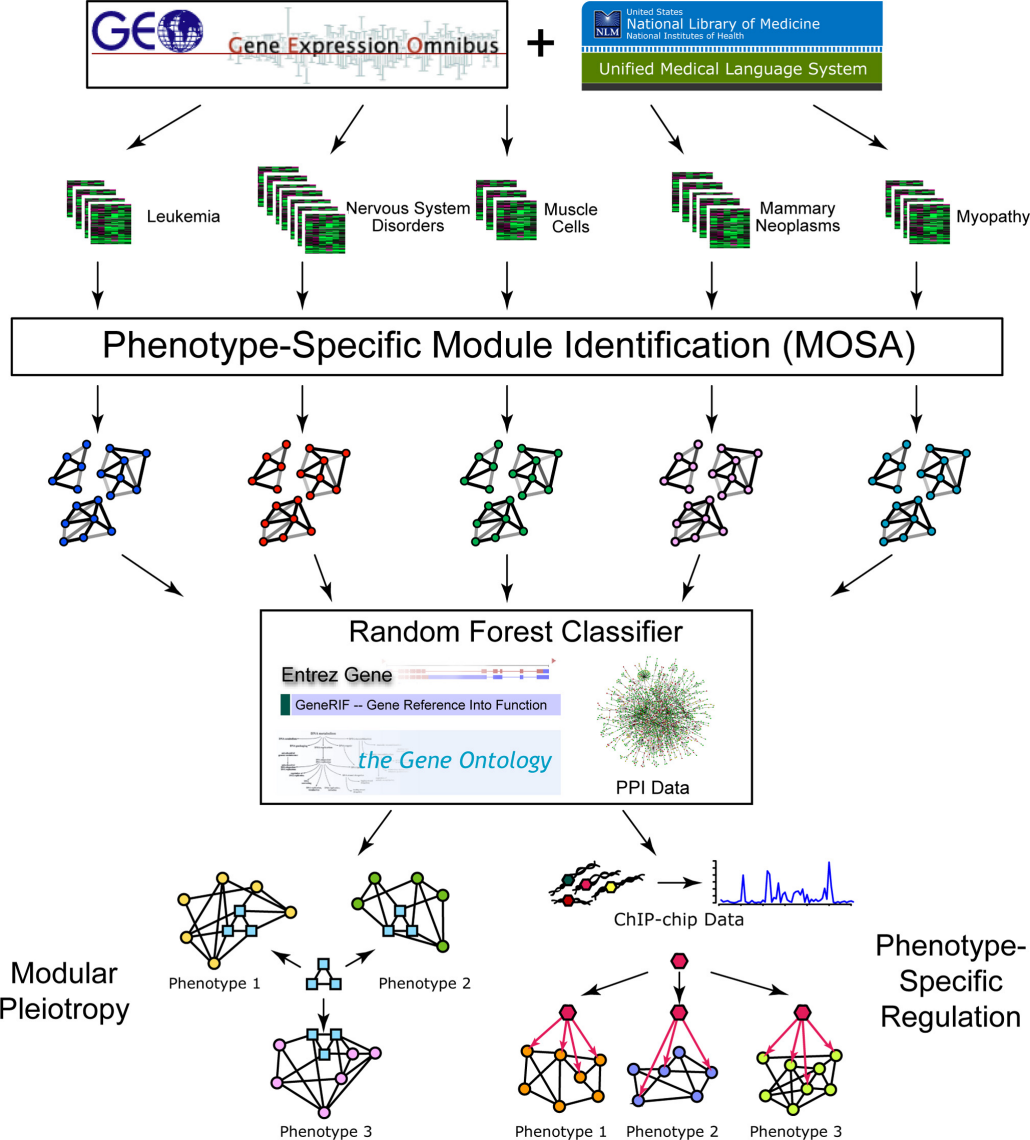
**A flow chart of our approach pipeline for each phenotype**. We designed a multiple objective simulated annealing (MOSA) algorithm to identify phenotype-specific coexpression modules in microarray datasets. We incorporated additional data sources into a random forest classifier to make novel gene-phenotype predictions. These modules and predictions were used to study human pleiotropy and phenotype-specific transcription regulation.

Our approach identified phenotype-specific modules preferentially coexpressed in microarray datasets that study particular phenotypes, and we used these modules to perform systematic phenotype prediction, study gene pleitropy, and integrated phenotype-specific transcription factor binding data to build dynamic regulatory networks (Figure 1). We collected 338 human microarray datasets from the NCBI Gene Expression Omnibus (GEO) [18]. By mapping the dataset annotations onto UMLS [19] phenotype terms we obtained 283 phenotype classes each of which contained at least 3 microarray datasets. For the purposes of this paper, we employ a broad interpretation of the term phenotype, which includes diseases, tissues, and cell types.

We previously designed a Multiple Objective Simulated Annealing (MOSA) algorithm that robustly identifies groups of genes that are preferentially coexpressed in datasets of a specific phenotype class [20]. The simulated annealing procedure was designed to optimize four characteristics of phenotype-specific coexpression modules: size, density, specificity for the phenotype datasets, and a summary statistic of module density which we term density differential. The goal of this approach was to identify genes modules that exhibit a coexpression signature which is specific to the phenotype in which they were identified, and therefore are likely to represent processes inherent to the phenotype.

Applying this approach, we identified 193,145 phenotype-specific coexpression modules that met our criteria of a minimum size of 7, minimum density of 0.66, and a minimum phenotypic enrichment *p*-value of less than 0.01 after FDR correction. These modules spanned 178 phenotypes and had an average size of 13.7. A more detailed description of this algorithm is outlined in the methods section.

In this study, starting with the phenotype-specific modules, we predicted gene phenotype associations by additionally incorporating the following three data sources: our database of gene-phenotype associations obtained from text-mining of the Gene Reference Into Function (GeneRIF) resource, the Gene Ontology database, and protein-protein interaction data (details in methods).

We trained a different random forest classifier for the 52 phenotypes for which the phenotype-specific coexpression modules contained genes with GeneRIF entries with the same phenotype. Each classifier therefore depended on the the set of genes that were known to be associated with the training phenotype via GeneRIF text mining, to which we refer to as *G*_*p*_. Our model contained 5 predictor variables, and contained one observation for each gene in the phenotype-specific modules. If a gene appeared in more than one module, the predictor variables were averaged across the modules.

Given a previously unannotated gene *g*_*i*_, which is a member of module *m*_*pj *_(the *j*^*th *^module specific to phenotype *p*), we predicted whether *g*_*i *_is associated with phenotype *p *by considering the following predictive features: i) the enrichment of the members of *m*_*pj *_for genes in *G*_*p*_; ii) the degree of GO annotation similarity between *g*_*i *_and *m*_*pj *_∩ *G*_*p*_; iii) and the number of protein-protein interactions between *g*_*i *_and *m*_*pj *_∩ *G*_*p*_. The first predictor variable was the negative log *p*-value of the hypergeometric test for enrichment of genes in the module for genes in *G*_*p*_. The second predictor variable was derived from protein-protein interaction (PPI) data. The PPI score was calculated by summing the total number of protein-protein interactions between the current gene and genes from *G*_*p *_in the same module. The remaining three predictor variables were derived from the three subtrees of GO (biological process, cellular component, and molecular function) as follows. For a pair of genes in the same module, the GO score for a subtree was calculated by first identifying all GO terms shared between the two genes. Once this set of distinct matching terms was established, the GO score was calculated by summing up the negative log *p*-values of the significance of matches to genes in *G*_*p*_.

**Figure 2:**
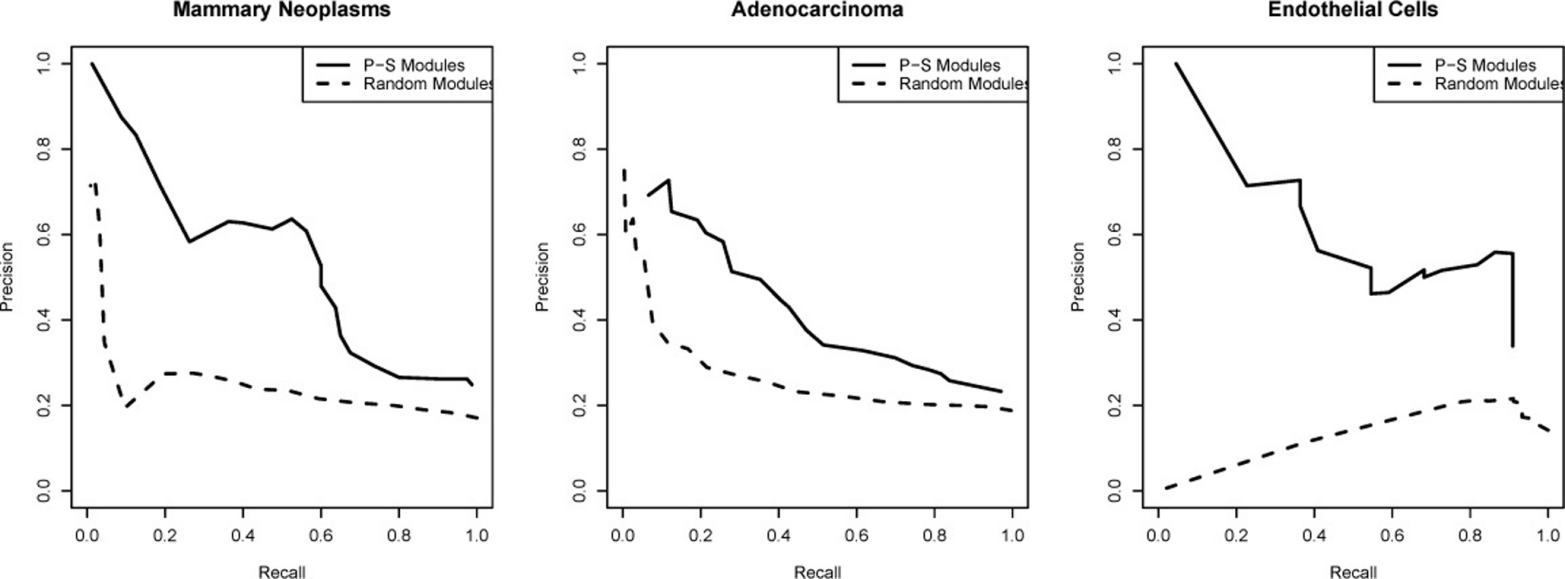
**Random forest performance**. Precision-recall plot depicting the training performance of the random forest classifiers for three phenotypes.

These classifiers trained on the predictive features discussed above predicted 6,558 gene-phenotype associations covering 3,183 genes. For a cumulative recall (including all phenotypes) of at least 20%, the precision of our predictions was approximately 65%. As the stringency of the cutoff parameter increases the precision continues to climb above 80%. This indicates that if we restrict the classifiers to a small number of predictions they become extremely accurate, which would be an ideal set to pursue in a clinical setting. The precision-recall plot, which differs from a ROC plot by only plotting statistics related to positive predictions, for three classifiers compared to randomly generated modules is shown in Figure 2. Each of our predictions is scored based on the fraction of classification trees from the random forest that voted for the prediction. Table 1 shows the predictions with the best scores for the 10 highest scoring predictions, stratified by phenotype, along with supporting evidence from published papers and Gene Ontology. GeneRIF does not contain all published papers that discuss a gene's association with a phenotype, so the published papers listed in this table represent information not used by the classifier. The first prediction is ADRB3 for the phenotype "Urologic Diseases." The potential for this gene to be involved in urologic diseases is supported by its presence in the human urinary bladder urothelium, as well as its regulation of urinary function [21]. Five of the predictions in this table are for phenotypes related to the immune system, such as "Phagocytes," "Inflammation," "Lymphocyte," "monocyte," and "Bone Marrow Cells." Many of these predictions are supported by both literature and their GO annotations which include myeloid cell differentiation, inflammatory response, and immune response. Two other interesting predictions are the chemokine genes CXCL11 and CXCL2 for the phenotypes "Bone Marrow Cells" and "Inflammation" respectively. Chemokines are master controllers of the migration of leukocytes, which originate in bone marrow and directly effect many functions related to the immune system including inflammation. The remaining predictions shown in the table are related to cancer and epithelial tissue related phenotypes.

**Table 1:** Highest confidence gene-phenotype association predictions stratified by phenotype.

Phenotype	Cutoff	Gene	Gene Description	PMID	Relevant GO Term
Urologic Diseases	1.00	ADRB3	adrenergic, beta-3-, receptor	18311486	
Phagocytes	1.00	PML	promyelocytic leukemia		myeloid cell differentiation
Genital Neoplasms, Female	1.00	EXO1	exonuclease 1	15328369	DNA repair
Inflammation	1.00	CXCL2	chemokine (C-X-C motif) ligand 2	12892904	inflammatory response
skin disorder	0.99	SC4MOL	sterol-C4-methyl oxidase-like		
Lymphocyte	0.99	FPRL1	formyl peptide receptor 2	15625007	G-protein coupled receptor protein signaling pathway
monocyte	0.99	TAP2	transporter 2, ATP-binding cassette, sub-family B (MDR/TAP)	12234057	immune response
Bone Marrow Cells	0.99	CXCL11	chemokine (C-X-C motif) ligand 11	15102366	immune response
Epithelium	0.98	ITGB4	integrin, beta 4	15194479	cell adhesion
Neoplasms	0.98	HMHA1	histocompatibility (minor) HA-1	14502255	intracellular signaling cascade

Each phenotype is listed with the predicted gene, the gene's description, and the proportion of decision trees from the random forest classifier that predicted its association to the phenotype. The final two columns provide supporting evidence in the form of a PubMed ID for a published work or a Gene Ontology annotation that is consistent with the phenotype.

To provide a more comprehensive analysis of our predictions, rather than just highlighting the highest scoring predictions, we performed functional enrichment analysis on each predicted set of associated genes for each phenotype. This analysis revealed that the majority of phenotype enriched biological processes are consistent with the functionality of the phenotype, which supports the quality of our predictions. For example, in the phenotype "Squamous cell carcinoma," our predicted genes are enriched for GO annotations related to skin cancer such as DNA replication, keratinization, and epidermis development. GO annotations are also consistent with the phenotypes for tissues. The phenotype "Brain" is enriched for synaptic transmission and monovalent inorganic cation transport. We highlight additional examples of significantly (FDR *p*-value < 0.01) overrepresented GO terms within predicted gene sets for phenotypes in Table 2.

**Table 2:** Overrepresented GO terms in predicted genes Seven of the UMLS phenotypes are highlighted, along with the number of phenotype-specific coexpression modules and number of novel predicted associated genes. The final column contains a subset of the overrepresented GO biological processes in the predicted genes that are consistent with the phenotype. The full table is available on our supplementary website.

Phenotype	Modules	Predictions	Over-represented GO annotations
Adenocarcinoma	2367	69	cell cycle process (3.5e-06) DNA replication (4.1e-06) cell cycle phase (8.5e-06)
Bone Marrow Cells	5390	421	immune response (3.8e-06) immune system process (7.2e-06) response to virus (2.0e-05)
Brain	8373	329	synaptic transmission (3.8e-06) monovalent inorganic cation transport (1.7e-05)
Connective and Soft Tissue Neoplasm	1463	22	cell cycle process (4.3e-14) cytoskeleton-dependent intracellular transport (8.6e-07)
Musculoskeletal Diseases	5421	324	actin filament-based process (8.2e-06)
Squamous cell carcinoma	1268	54	DNA replication (2.5e-08) keratinization (8.1e-05) epidermis development (1.0e-04)
nervous system disorder	8235	628	respiratory electron transport chain (5.1e-07) acetyl-CoA catabolic process (3.2e-05)

### The prevalence of gene pleiotropy

Our large-scale gene-phenotype association prediction provided us with a unique opportunity to systematically study gene pleiotropy. The simplest definition of pleiotropy, a gene being annotated with multiple UMLS phenotype terms, is inadequate here. For example, TAL1 is annotated with both "leukemia" and "Immunoproliferative Disorders," but these do not constitute two distinct phenotype associations because one is simply a more general phenotype than the other. Other groups have employed phenotype distance metrics and declared two phenotypes different when their distance exceeded a certain threshold [10,22]. However, this type of method relies on the selection of an arbitrary threshold, which can significantly affect the results. Here, we defined pleiotropy using the structure of the parental links within the UMLS phenotype ontology. We considered a gene pleiotropic if it was annotated with at least two phenotypes, neither of which is a descendant of the other.

Applying this definition of pleiotropy to only the disease phenotypes revealed that 40.9% of the 8,504 genes in our study are associated with at least one pair of distinct phenotypes. It should be noted that this percentage is likely to be an over estimate of pleiotropic genes, since UMLS text mining of GeneRIF terms can lead to incorrect annotations and therefore pleiotropy. The addition of our phenotype predictions resulted in 607 novel pleiotropic genes. Furthermore, 725 genes that were previously pleiotropic were annotated with a new phenotype distinct from all previous annotations, resulting in a new case of pleiotropy.

**Figure 3:**
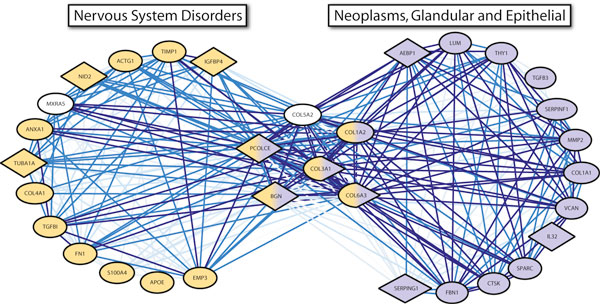
**An example of modular pleiotropy with a pleiotropic component related to the extracellular matrix**. The modules shown are specific to microarray datasets that study "nervous system disorders," and "Neoplasms, Glandular and Epithelial." The shaded elliptical nodes represent genes associated with the module phenotype (via GeneRIF text mining), whereas the shaded diamond nodes represent predictions made by our random forest classifier. The edge opacity indicates how often a pair of genes was coexpressed in the microarray datasets for which the module was coexpressed.

Pleiotropy can be determined and observed in a variety of ways. In this study, we predicted whether a particular gene exhibits pleiotropic behavior based upon its membership in phenotype-specific coexpression modules. We therefore term this type of pleiotropy *modular pleiotropy*, in which a gene's pleiotropic behavior is determined by module membership. An example that illustrates this phenomenon is depicted in Figure 3 involving two modules, one specific to "nervous system disorders" and the other to "Neoplasms, Glandular and Epithelial." These modules share six genes related to the extracellular matrix, the substrate upon which cells migrate, proliferate, and differentiate. It is not surprising to find these genes associated with epithelial cancer, as many studies have shown that the extracellular matrix is heavily involved in tumor progression and metastasis [23,24]. The extracellular matrix also plays a major role in the development and repair of the central nervous system, supporting its presence in both modules [25]. In each of the two overlapping modules, the remaining, unshared genes also include genes from the extracellular matrix and additional genes that are highly specific to the phenotypes in which the modules were discovered. These include the well known Alzheimer's susceptibility locus APOE, an actin gene differentially expressed in schizophrenia patients (ACTG1), a brain-specific tubulin associated with behavioral defects (TUBA1A), and a tubulin polymerizer that serves as a neuroprotectant (S100A4). The neoplasms module also contains genes related to the extracellular matrix, and genes with known roles in neoplastic processes such as tumor progression, tumor necrosis induction, tumor suppression, and tumor invasion [26,27].

We would also like to highlight an interesting prediction of pleiotropy for the gene BGN, which is present in both modules. This prediction is supported by the RefSeq entry for this gene, which states that it is thought to transfer growth factors between cells and that it may promote neuronal survival. Some additional evidence for BGN's activity in cancer was discovered by a study that showed BGN controls cell growth in pancreatic cancer cells [28]. Studies have demonstrated BGN's potential role in the human nervous system as well, as BGN is overexpressed in rats after brain injury and it sustained the survival of rat neocortical neurons in culture [29,30].

The above example nicely illustrates the power of our classification method to make reliable phenotype predictions, and in particular to identify pleiotropy and its modular context.

### Phenotype-specific transcriptional regulation

We hypothesized that regulatory networks are not static relationships between transcription factors and their target genes, but rather dynamic networks that vary to dictate different observed phenotypes, such as tissue types and diseases. Thanks to the accumulation of ChIP-chip experiments, each of which provides genome-wide TF binding data derived under particular phenotypic conditions, we were able to test this hypothesis by determining whether TF binding detected under a phenotypic condition preferentially occurs in genes belonging to modules specific to the same phenotype. This concordance between or phenotype-specific coexpression modules and phenotype-specific binding from ChIP-chip data also serves to independently validate the phenotype-specificity of our modules.

We compiled 77 ChIP-chip datasets from the public repositories and also manually collected results from literature publications. These data include 69 TFs and 16,122 target genes, which can be found on our online Phenotype Prediction Database [1]. Applying our text mining procedure to these datasets resulted in a total of 208 phenotypes including diseases, tissues and cell types. Of these 208 phenotypes, we focused on the 97 phenotypes that were also studied by the microarray datasets we had collected. This provided us with one of the most comprehensive collections of regulatory data available, with the phenotypic conditions that each dataset studied.

For each phenotype-specific module, we tested the member genes for enrichment of TF binding derived from ChIP-chip datasets that study related phenotypes. Out of the 97 ChIP-chip phenotypes, 43 exhibited a statistically significant preferential binding (Mann-Whitney test, FDR < 0.05), indicating that target genes from ChIP-chip data are more likely to form coexpression modules in microarray datasets whose phenotype annotations match those of the ChIP-chip experiment. The full table of *p*-values resulting from this analysis are available online on our Phenotype Prediction Database [1].

**Figure 4:**
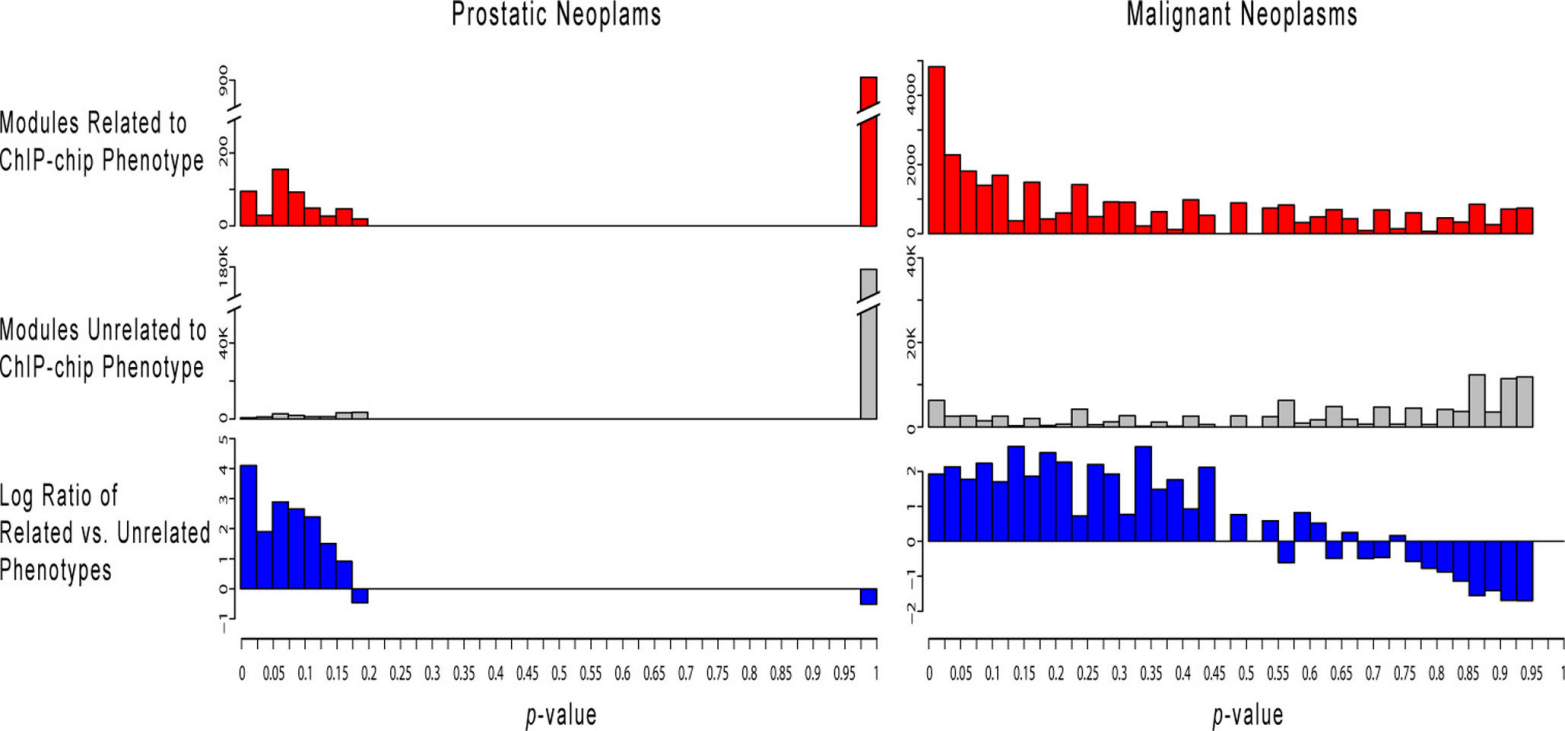
**Distribution of ChIP-chip TF binding enrichment *p*-values for phenotype-specific coexpression modules**. The distributions of ChIP-chip TF-binding enrichment *p*-values are shown for two ChIP-chip phenotypes: Prostatic Neoplasms and Malignant Neoplasms. The first and second rows of distributions correspond to those derived from microarray datasets with a phenotype *related *to the ChIP-chip data phenotype and those derived from *unrelated *phenotypes respectively. The final row displays the log_2 _ratio of the two distributions relative to their respective total sizes.

One example of a phenotype that exhibits preferential ChIP-chip binding is "Prostatic Neoplasms," which is studied by a single ChIP-chip dataset profiling androgen receptor binding. The phenotype-specific preferential binding is shown in Figure 4. The proportion of phenotype-specific modules for this phenotype that are significantly enriched for androgen receptor binding (hypergeometric *p*-value < 0.025) is over 16 times higher than the modules specific to other phenotypes. This strong over representation of significant binding enrichment for our modules demonstrates the ability of our method to detect evidence of phenotype-specific TF binding, despite being provided only a small fraction of the complete transcriptional regulatory information related to this phenotype.

**Figure 5:**
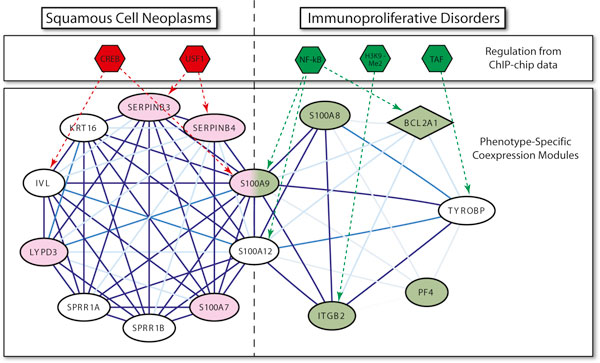
**An example of modular pleiotropy with phenotype-specific regulatory data**. The genes in the module on the left are specifically coexpressed in "Squamous Cell Neoplasms" microarray datasets, while those on the right are specifically coexpressed in "Immunoproliferative Disorders" microarray datasets. Two phenotype-specific modules containing genes with GeneRIF entries for the same phenotype (shaded). The edge opacity indicates how often a pair of genes was coexpressed in the microarray datasets for which the module was coexpressed. The opacity the edges indicates the degree of coexpression. The transcription factor binding, derived from ChIP-chip datasets that studied phenotypes related to the microarray datasets, is shown with dotted arrows.

The second example in the figure is the more general phenotype "Malignant Neoplasms". The distribution of TF binding enrichment is more continuous for this example due to the larger number of related phenotype-specific modules and ChIP-chip datasets. As with the previous example, there is a higher relative frequency of significantly bound modules specific to phenotypes that match the phenotype of the TF binding. Also, the frequency of the most significant binding enrichment (hypergeometric *p*-value < 0.025) within the distribution of the related phenotype-specific modules is more than twice as high as the frequency of all other enrichment *p*-values. This can be attributed to the much higher quantity of ChIP-chip data available for this phenotype, which allowed for more significant binding for our transcription modules. This result implies that as more ChIP-chip data becomes available, we would expect to see the significance of binding enrichment of our phenotype-specific modules continue to increase. Although the complete set of phenotype-specific binding is not available, we were still able to construct portions of these dynamic transcriptional networks by combining phenotype-specific coexpression modules with TF binding from the same phenotypes. For example, Figure 5 shows two overlapping modules, each specific to different phenotypes, and each significantly enriched for phenotype-specific binding that matches its phenotype. The two modules are each preferentially coexpressed in datasets studying a specific phenotype: one "Squamous Cell Neoplasms" and the other "Immunoproliferative Disorders."

As with the previous example of pleiotropy, these two modules are phenotype specific and are enriched with genes related to each phenotype. The "Squamous Cell Neoplasms" module contains ten genes, five of which were previously annotated with the phenotype, and is also significantly enriched for GO terms consistent with cancer of squamous tissue: keratinocyte differentiation (*p*-value 2.8e-9) and epidermis development (*p*-value 5.1e-7). The seven gene "Immunoproliferative Disorders" module contains four genes that were annotated with the phenotype, as well as being enriched for GO annotations related to the immune system such as leukocyte chemotaxis (*p*-value 5.1e-7) and defense response (*p*-value 5.0e-6). The modules share two pro-inflammatory calcium binding genes, S100A9 and S100A12, that are implicated together in a number of diseases [31]. S100A9 was previously annotated with both phenotypes, making it an example of a pleiotropic gene confirmed by its presence in two coexpression modules.

Our analysis of phenotypic conditions of the ChIP-chip data revealed that each of these modules were significantly enriched for phenotype-specific TF binding. The binding for the "Squamous Cell Neoplasms" module was derived from 18 ChIP-chip datasets that study the related phenotype "Epithelial Cells." The module is significantly enriched for the USF1 transcription factor (*p*-value 0.02) which binds SERPINB3 and SERPINB4 in epithelial cells, as well as marginally enriched for CREB (*p*-value 0.06) which binds IVL and S100A9.

The binding for the 'Immunoproliferative Disorders" module was derived from datasets that study "Myeloid Cells," which was studied by 16 datasets. The majority of the binding was by the transcription factor NF-*k*B, which in myeloid cells binds three genes (*p*-value 0.0006) and is known to play a role in regulation of the immune system as well as types of cancer [32]. This included both shared genes (S100A9, S100A12), and a gene that performs cytoprotective and anti-apoptotic roles in neutrophils (BCL2A1) [33]. This concordance between microarray expression data and regulatory ChIP-chip data demonstrates that our approach of finding phenotype-specific transcription modules can not only identify gene-phenotype relationships, but that it can be combined with TF binding data to further explain the molecular mechanisms and regulatory pathways underlying phenotypes and pleiotropy.

## Conclusion

We have developed a method to determine the phenotypic effects of particular genes in humans, where phenotype prediction is far more difficult than in model organisms. This difficulty is largely due to our inability to study the underlying biological pathways directly, for example by inducing mutations or otherwise perturbing phenotype-related pathways. Our approach circumvents this limitation because the identified gene modules are preferentially active in datasets that study a particular phenotype, and thus are likely to represent phenotype-specific pathways. Using public resources that provide phenotype data, such as UMLS and GeneRIF, we made novel predictions that we believe will be useful in a clinical setting, further our understanding of disease pathways, and serve as potential drug targets.

In addition, we propose that regulatory networks should be viewed as dynamic networks with topology dependent upon the phenotypic conditions that they model. Since ChIP-chip experiments examine binding data under specific phenotypic conditions, their data provide a static snapshot of this dynamic regulatory network. Our results support this hypothesis by demonstrating preferential binding of transcription factors to coexpression modules in similar phenotypes.

Our pleiotropy analysis, complemented by our phenotype predictions, indicates that pleiotropy in humans may be a more widespread phenomenon than previously thought. Further study of how coexpression partners change in different tissues and the role phenotype-specific transcriptional regulation plays in shaping these changes will provide deeper insight into the underlying cause of phenotypic diversity as well as genetic pleiotropy.

## Methods

### Data sources

The microarray datasets were downloaded from NCBI GEO, and were from Affymetrix human platforms U95, U133, U133A 2.0, and U133 Plus 2.0. All datasets from these platforms with at least eight experimental samples were included, for a total of 338 datasets, which are included in the online Phenotype Database [1]. We only included the 8,504 genes that were present in all datasets and had at least one probe that mapped uniquely to it. We annotated each of the datasets with phenotype terms by mining the titles and MeSH terms of the associated papers with the UMLS MMTx tool, following the approach by Butte and Kohane [34].

### Creation of a database of GeneRIF gene-phenotype associations

To objectively assign individual genes to the phenotypes with which they are known to be associated, we used the UMLS MetaMap Transfer tool to identify phenotypic terms from the UMLS Metathesaurus that were present in the raw text for each gene within Gene Reference Into Function (GeneRIF). GeneRIF submissions often contain concise information about the function of the gene, or whether it is known to be associated with any disease phenotypes. Therefore, by applying the UMLS MetaMap Transfer tool, we created a database of gene-phenotype associations derived from literature sources. Each association in the database was traced back to the root of the tree defined by the *parental *phenotypes described in the next section.

### Phenotype relationships

For the Phenotype-Prediction and ChIP-chip analysis we defined a set of *related *phenotypes for each phenotype using the relationships provided by the UMLS Metathesaurus. The *related *phenotypes were defined by immediate neighbors using the set of relationships {RB, RL, RN, RO, RQ, RU, SY}. The relationships used represent different types of similarity as well as whether two phenotypes are simply synonymous. The *parental *phenotypes were defined using the "PAR" relationships from the UMLS Metathesaurus. For a given phenotype, all phenotypes present in the trace to the root of the UMLS graph defined by these "PAR" edges were considered *parental *phenotypes.

### Gene module detection

Our procedure is explained in detail in our previous paper [20]. For completeness, we present an overview here. The goal of the algorithm we used is to find sets of genes that are significantly coexpressed specifically in datasets related to particular phenotypes. For each of the 338 datasets in our study, we constructed an unweighted coexpression network composed of the top 150,000 correlated genes. We designed a multi-objective simulated annealing algorithm, with four objective functions designed to maximize the size, absolute density, relative density (to non-phenotype datasets), and specificity of the coexpression module for the phenotype datasets. The purpose of the relative density, or density differential, was to reward subtle changes in module density that would not be detected by the phenotype-specificity objective function. The objective functions we designed are outlined below.

where

 is the set of datasets annotated with the current phenotype,

 is the set of datasets in which the gene cluster is dense,

and *Y *~ *hypergeometric *().

After completion of the simulated annealing procedure, we performed post filtering to remove low-quality modules. Our filtering criteria for each module was a minimum size of 7, minimum density of 0.66, and a minimum phenotypic enrichment *p*-value of less than 0.01 after FDR correction. These modules formed the basis for the phenotype prediction presented in this paper.

### Random forest training

The random forest classifier was trained using 500 trees and a maximum terminal node size of 10. The cross-validation statistics reported were calculated using the Out Of Bag (OOB) errors provided by the training process. For each classifier, the positive training set was composed of all genes that contained a GeneRIF entry that mentioned the training phenotype. The negative set is more difficult to define, since genes that are actually associated with a particular phenotype may not be labeled as such due to imperfect text processing or missing GeneRIF annotations. Therefore, to minimize the error of our classifier we used the phenotype relationships defined by the UMLS to define our negative training set. We only included genes in the negative set that contained GeneRIF entries that were neither *related *nor *parental *to the training phenotype (see Section Phenotype relationships for more detail). The remaining genes, which included the *related *and *parental *genes, and those with no GeneRIF entries, were excluded from the training procedure and were used for prediction after the training of each phenotype classifier. The final predictions reported for each phenotype were the positive predicted genes from our training, as well as all positively predicted genes from our prediction set.

### ChIP-chip phenotype specificity

The phenotypes studied by each ChIP-chip dataset was determined by performed text mining on the datasets' title, description, MeSH headings, and associated publications' title and abstract using the UMLS MMTx text mining tools. Given a single phenotype present in both ChIP-chip data and microarray data, we defined two sets of coexpression clusters: one derived from phenotypes *related *to the current phenotype, and the remaining modules *unrelated *to the current phenotype. We performed a Mann-Whitney test on the hypergeometric test *p*-values for TF enrichment in modules from related phenotypes versus the rest of the modules, to determine whether transcription factors from the phenotype ChIP-chip experiments exhibited significant preferential binding.

## Competing interests

The authors declare that they have no competing interests.

## Authors' contributions

MM, JNE, CD, MSW, and XJZ designed the study and drafted the manuscript. MM implemented the simulated annealing algorithm, and performed the phenotype prediction and the subsequent analyses. JNE contributed to the analyses. CD collected and analyzed the ChIP-chip data. XJZ supervised the project. All authors read and approved the final manuscript.
